# Aberrant HER3 ligand heregulin-expressing head and neck squamous cell carcinoma is resistant to anti-EGFR antibody cetuximab, but not second-generation EGFR-TKI

**DOI:** 10.1038/s41389-019-0164-9

**Published:** 2019-09-30

**Authors:** Kimio Yonesaka, Kaoru Tanaka, Mutsukazu Kitano, Hisato Kawakami, Hidetoshi Hayashi, Masayuki Takeda, Kazuko Sakai, Kazuto Nishio, Katsumi Doi, Kazuhiko Nakagawa

**Affiliations:** 1 Department of Medical Oncology, Kindai University Faculty of Medicine, Osaka-Sayamashi, Osaka Japan; 2 Department of Otolaryngology, Kindai University Faculty of Medicine, Osaka-Sayamashi, Osaka Japan; 3 Department of Genome Biology, Kindai University Faculty of Medicine, Osaka-Sayamashi, Osaka Japan

**Keywords:** Head and neck cancer, Cancer therapeutic resistance

## Abstract

The anti-epidermal growth factor receptor (EGFR) antibody cetuximab is standard therapy for head and neck squamous cell carcinoma (HNSCC). However, most HNSCC tumors are resistant to it and require alternative treatments. Here, we explored the mechanism of cetuximab resistance and evaluated its clinical relevance in HNSCC. An unbiased comprehensive transcriptome analysis was performed on cetuximab-resistant HNSCC FaDuCR cells. The causative resistance genome was knocked down with siRNA, cell signaling was immunologically analyzed, and drug efficacy was evaluated in vitro and in vivo. The mRNA in situ hybridization (ISH) of the causative genome was performed using 28 excised HNSCC tumors and its relationship with cetuximab efficacy was analyzed. FaDuCR cells were resistant to cetuximab, whereas parental FaDu cells were susceptible to it. FaDuCR cells expressed consistently higher levels of phosphorylated Akt than FaDu cells despite cetuximab exposure. A comprehensive transcriptome analysis revealed that the HER3-ligand heregulin was upregulated in FaDuCR cells compared to FaDu cells. Heregulin knockdown in FaDuCR cells repressed HER3 and Akt phosphorylation and recovered cetuximab anticancer efficacy. In contrast, pan-HER family tyrosine kinase inhibitors such as afatinib decreased HER3 and Akt phosphorylation in FaDuCR cells and inhibited FaDuCR tumor growth. Two of the 28 HNSCC tumor samples presented aberrant heregulin expression comparable to that of FaDuCR cells and were resistant to cetuximab therapy. In HNSCC, heregulin-mediated HER3-Akt activation causes resistance to cetuximab but not to second-generation EGFR-tyrosine kinase inhibitors. Subpopulations with aberrant heregulin-expressing HNSCC might be resistant to cetuximab.

## Introduction

Head and neck squamous cell carcinoma (HNSCC) is the seventh most commonly occurring cancer worldwide^1^. The prognosis of recurrent and/or metastatic HNSCC is very poor and the median survival is <12 mo^2^. The standard treatment for recurrent and/or metastatic HNSCC is a combination of the anti-epidermal growth factor receptor (EGFR) antibody cetuximab and platinum-based chemotherapy^2,3^. Anti-PD-1 therapy improved HNSCC prognosis, but patient responsiveness was only 13.3%^4^. To improve HNSCC prognosis, novel molecular targeted therapies are required.

EGFR is a member of the human epidermal growth factor receptor (HER) family of receptor tyrosine kinases and is implicated in HNSCC pathogenesis^5^. EGFR is overexpressed in ≤90% of HNSCC tumors compared with the levels found in normal mucosae. EGFR overexpression is correlated with poor prognosis^5–7^. The structural conformation of EGFR is altered when it binds with ligands such as EGF, TGF-α, amphiregulin, and others. It then undergoes dimerization, and its signaling pathway is activated^8,9^.

Cetuximab is an anti-EGFR monoclonal antibody targeting the extracellular EGFR domain. It interferes with the binding of EGFR to its ligands^10^. Previous studies have reported that cetuximab efficacy depends on the expression of the EGFR ligand (especially amphiregulin) in HNSCC, non-small cell lung cancer (NSCLC), and colorectal cancer (CRC)^10,11^. Cetuximab alone or in combination with chemotherapy may improve the prognosis of patients with HNSCC, NSCLC, and CRC^2,12–14^. However, cetuximab monotherapy showed only modest activity and a limited objective response rate of 13% in recurrent or metastatic HNSCC. In contrast, the cancer progressed in patients on platinum therapy^15^. All patients initially responding to cetuximab-based therapy eventually developed resistance to it. In approximately 40% of all CRC cases, the dominant resistance factor was a RAS/RAF gene mutation^11,16^. HER2 genomic amplification induces anti-EGFR-therapy resistance in CRC and NSCLC^17,18^. HER2-targeting therapy is being intensively investigated for NSCLC and CRC^19,20^. In HNSCC, however, the mechanism of cetuximab resistance is not fully understood. Its elucidation may help improve EGFR-targeting therapy in HNSCC.

An alternative EGFR-targeting strategy is the administration of low-molecular-weight tyrosine kinase inhibitors (TKIs). These compete with adenosine triphosphate (ATP), bind to the intracellular domain of the EGF receptor, and prevent any further activation of the intracellular signaling cascade^21^. A previous preclinical trial reported that EGFR-TKI had efficacy against HNSCC cell lines with aberrant EGFR ligand expression^10^. However, the first-generation EGFR-TKI gefitinib combined with chemotherapy did not improve survival of patients with HNSCC^22^. Trials with the first-generation EGFR-TKI erlotinib failed because of low accrual (NCT00448240, NCT00412217). In contrast, second-generation EGFR-TKIs irreversibly inhibit pan-HER family tyrosine kinases including EGFR, HER2, and HER4. However, the optimal subpopulation to be treated by second-generation EGFR-TKI therapy has not been determined^23,24^.

The aim of the present study was to elucidate the underlying mechanisms of cetuximab resistance in HNSCC in the attempt to improve the prognosis of patients with this form of cancer. To this end, we assessed the efficacy of second-generation EGFR-TKIs against a cetuximab-resistant HNSCC cell line model. We also performed mRNA-ISH on excised HNSCC tumors to evaluate the expression of the gene encoding cetuximab resistance.

## Results

### Cetuximab-resistant HNSCC FaDuCR cells maintain Akt activation under cetuximab exposure

**Fig. 1:**
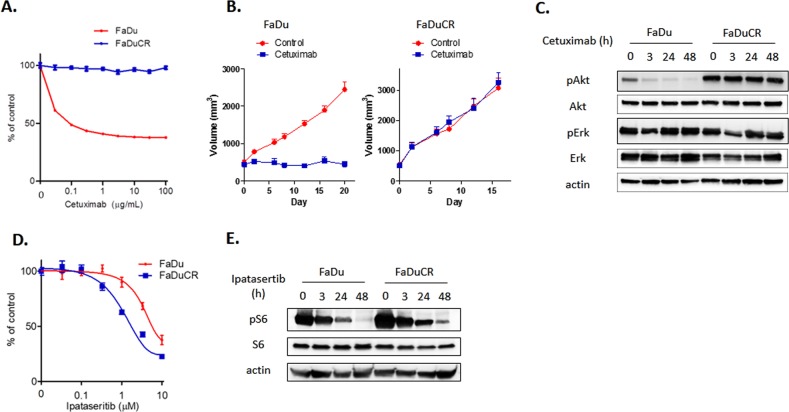
Anti-cancer efficacy of cetuximab in FaDu and FaDuCR cells. **a** In vitro growth inhibition assay of cetuximab. FaDu or FaDuCR cells were treated with the indicated concentrations of cetuximab for 5 d. Cell viability was evaluated using the CellTiter-Glo assay and is shown relative to untreated control cells (mean ± SD of six independent experiments). **b** In a mouse FaDu or FaDuCR xenograft model, tumors were treated with cetuximab 40 mg kg^−1^ or vehicle twice per week. Tumor growth curves are shown. Each group consisted of 10 mice. Data are mean ± SEM. **c** FaDu or FaDuCR cells were treated with 10 ng mL^−1^ cetuximab for up to 48 h and probed for the indicated proteins. **d** In vitro growth inhibition assay of ipatasertib. FaDu or FaDuCR cells were treated with the indicated concentrations of ipatasertib for 5 d. Cell viability was evaluated using the CellTiter-Glo assay and is shown relative to untreated control cells (mean ± SD of six independent experiments). **e** FaDu or FaDuCR cells were treated with 3 µM ipatasertib for up to 48 h and probed for the indicated proteins

We established a cetuximab-resistant HNSCC cell line. Cetuximab-sensitive FaDu HNSCC cells were treated with increasing concentrations of cetuximab (up to 10 μg mL^−1^) for 6 mo. The cells freely proliferated in medium containing 10 μg mL^−1^ cetuximab and were named FaDuCR. An in vitro growth inhibition assay demonstrated that the viability of the parent FaDu cells decreased with increase in cetuximab concentration, whereas FaDuCR cells continued to proliferate even at 100 μg mL^−1^ cetuximab (Fig. 1a). We also evaluated cetuximab efficacy in FaDu- or FaDuCR-xenografted mouse models. Both cell types were implanted in nude mice. When the tumor volumes reached 200 mm^3^, the mice were divided into groups of 10 and administered either 40 mg kg^−1^ cetuximab or vehicle by intraperitoneal injection. The FaDu xenograft tumors did not grow under cetuximab treatment (Fig. 1b). On the contrary, the FaDuCR xenograft tumors continued to grow in the presence of cetuximab at a rate equivalent to that of the vehicle control (Fig. 1b).

We analyzed signaling in FaDu and FaDuCR cells by immunoblotting. The cells were cultured in medium containing 10 μg mL^−1^ cetuximab, and then harvested at the indicated time points. The treated FaDu cells maintained ERK phosphorylation but exhibited decreased Akt phosphorylation over time (Fig. 1c). In contrast, FaDuCR cells maintained both ERK and Akt phosphorylation despite 48 h of cetuximab exposure (Fig. 1c). Akt phosphorylation was higher in the unexposed FaDuCR cells than in the unexposed FaDu cells. To determine whether FaDu and FaDuCR cells depended on the Akt pathway for proliferation, we performed an in vitro growth inhibition assay using the Akt-specific inhibitor ipatasertib. This treatment decreased the viable count of both FaDu and FaDuCR cells in a concentration-dependent manner (Fig. 1d). FaDuCR cells were more sensitive to ipatasertib than the parental FaDu cells (IC_50_ = 0.7 and 3.3 μM, respectively). An immunoblotting assay revealed that ipatasertib decreased S6 phosphorylation downstream of the Akt pathway in both FaDu and FaDuCR cells in a time-dependent manner (Fig. 1e). Therefore, the parent FaDu cells require the EGFR-Akt pathway to proliferate, and cetuximab inhibits FaDu proliferation by blocking this pathway. On the contrary, FaDuCR cells were resistant to cetuximab because Akt activation is independent of EGFR activation and is maintained by an unknown secondary mechanism.

### Ligand-dependent HER3-Akt signaling activation causes cetuximab resistance in FaDuCR cells

**Fig. 2:**
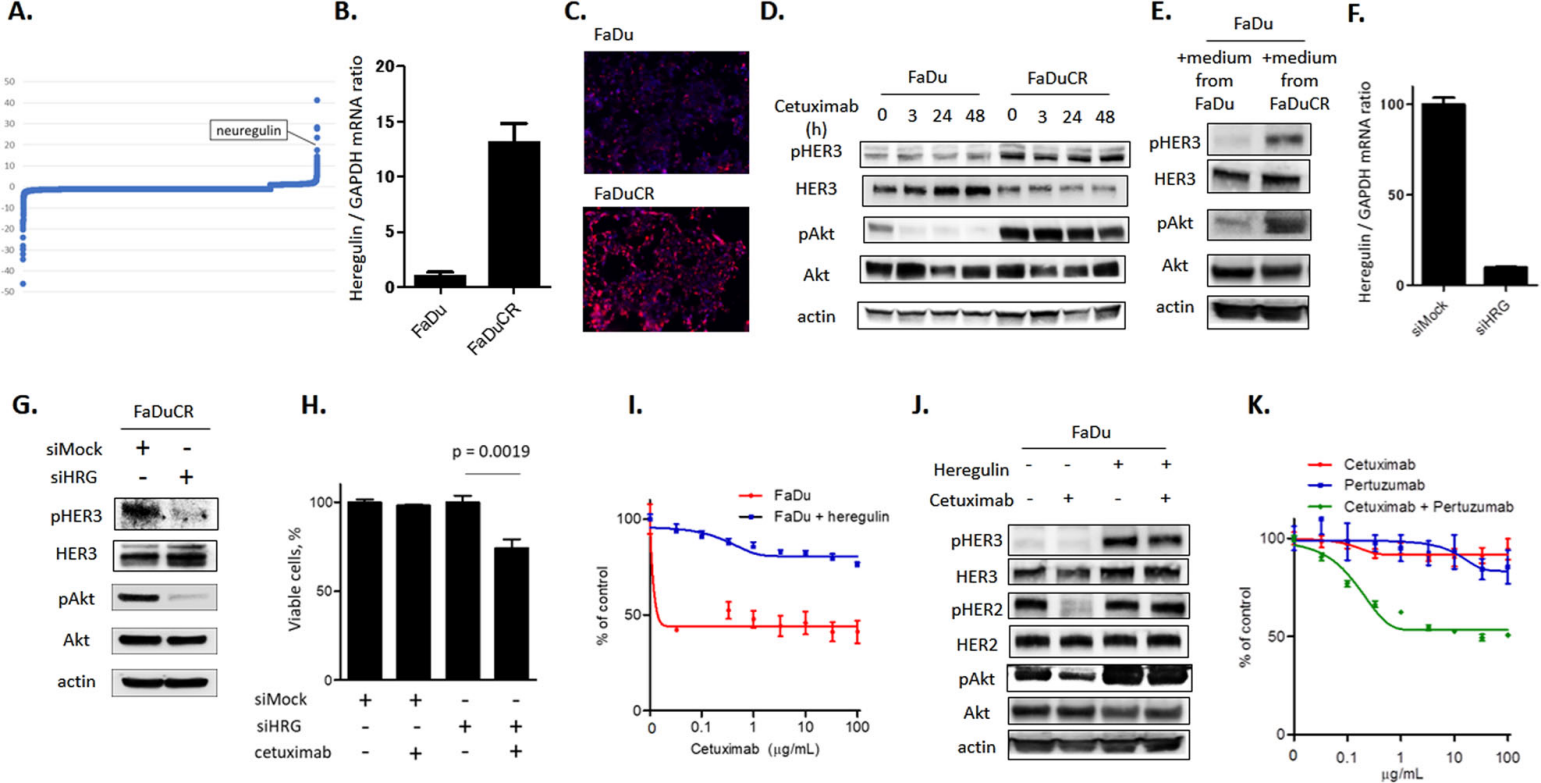
Heregulin-dependent HER3-Akt signaling activation causes cetuximab resistance in FaDuCR cells. **a** Comprehensive transcriptome analysis to evaluate alterations in FaDuCR cells relative to those in FaDu cells. X-axis: genomes. Y-axis: fold-change in gene expression in FaDuCR compared with that in FaDu. **b** mRNA was obtained from FaDu and FaDuCR cells and heregulin mRNA expression was quantitatively analyzed by PCR with GAPDH normalization (mean ± SD of three independent experiments). **c** Heregulin mRNA was revealed by in situ hybridization for FaDu and FaDuCR cells. **d** FaDu or FaDuCR cells were treated with 10 ng mL^−1^ cetuximab for up to 48 h and probed for the indicated proteins. **e** Culture medium of FaDu cells and FaDuCR cells was harvested and exposed to FaDu cells for 1 h. These cells were harvested and probed for the indicated proteins. **f** FaDuCR cells were transiently transfected with siRNAs targeting heregulin (HRG) or mock control. Heregulin mRNA expression was quantitatively analyzed by PCR with GAPDH normalization (mean ± SD of three independent experiments). **g** FaDuCR cells were transiently transfected with siRNAs targeting HRG or mock and probed for the indicated proteins. **h** FaDuCR cells were transiently transfected with siRNAs targeting HRG or mock and treated with 10 ng mL^−1^ cetuximab for 72 h. Cell viability was evaluated using the CellTiter-Glo assay and is shown relative to that of untreated control cells (mean ± SD of six independent experiments). **i** FaDu cells were treated with the indicated cetuximab concentrations for 5 d in the presence of 50 ng mL^−1^ heregulin in the culture medium. Cell viability was evaluated using the CellTiter-Glo assay and is shown relative to that of untreated control cells (mean ± SD of six independent experiments). **j** FaDu cells were treated with or without 10 ng mL^−1^ cetuximab in the presence or absence of 50 ng mL^−1^ heregulin in the culture medium for 1 h and probed for the indicated proteins. **k** FaDuCR cells were treated with the indicated concentrations of cetuximab, pertuzumab, and both drug combination for 5 d. Cell viability was evaluated using the CellTiter-Glo assay and is shown relative to that of untreated control cells (mean ± SD of six independent experiments)

We performed a comprehensive genomic analysis to elucidate the secondary mechanism of Akt activation in FaDuCR cells. A comprehensive NGS-based cancer panel failed to detect new mutations in FaDuCR cells (data not shown). However, a comprehensive transcriptome analysis revealed alterations in the expression of a part of the genome in FaDuCR cells compared with that in FaDu cells (Fig. 2a, Table 1). The HER family ligand gene *NRG1* encoding heregulin was among the 10 most highly upregulated genes. The quantitative PCR assay revealed approximately 13× higher heregulin expression in FaDuCR cells than in FaDu cells (*P* = 0.0003; Fig. 2b). mRNA in situ hybridization (ISH) also detected significantly higher heregulin mRNA expression in FaDuCR cells than in FaDu cells (12.9 and 3.4 times, respectively; Fig. 2c). As heregulin is a ligand for HER3, we used immunoblotting to analyze the levels of HER3 and its phosphorylation in FaDu and FaDuCR cells treated with cetuximab for up to 48 h. FaDuCR cells expressed HER3 phosphorylation at a higher level than that of FaDu cells and maintained it under 48 h cetuximab exposure (Fig. 2d). However, the total HER3 level was lower in FaDuCR cells than in FaDu cells. Therefore, the observed relative increase in HER3 phosphorylation in FaDuCR cells was caused by heregulin upregulation rather than HER3 augmentation. FaDuCR cells expressed higher Akt phosphorylation level than FaDu cells as well as maintained it and HER3 phosphorylation under prolonged cetuximab exposure (Fig. 2d). Unlike FaDuCR cells, the EGFR-dependent parental FaDu cells presented downregulated Akt phosphorylation and no HER3 alteration after extended cetuximab exposure (Fig. 2d). Furthermore, we measured the secreted amount of heregulin in the culture medium of both FaDu and FaDuCR cells using the ELISA. The heregulin level was under the detection limit (82.3 pg mL^−1^) in the culture medium of FaDu cells, whereas its level (2495 pg mL^−1^) was higher than that of FaDuCR cells (Supplementary Fig. 1A). We then examined whether the culture medium of FaDu or FaDuCR cells can activate the HER3 and Akt pathways in FaDu cells. FaDu cells were exposed to the culture medium for 1 h, and then harvested for immunoblotting. The culture medium of FaDuCR cells increased HER3 and Akt phosphorylation in FaDu cells compared with that of medium of FaDu cells (Fig. 2e). These results suggest that heregulin upregulation may activate the HER3-Akt pathway in FaDuCR cells as an alternative to the EGFR-Akt pathway.

**Table 1:** The abnormal expression of a part of the genome in FaDuCR cells compared with that in FaDu cells

Groups	Gene symbol	Description	Fold change
10 most downregulated genes	KRT4	Keratin 4, type II	−46.17
	SLPI	Secretory leukocyte peptidase inhibitor	−34.45
	TNFSF10	Tumor necrosis factor (ligand) superfamily, member 10	−31.87
	KRT13	Keratin 13, type I	−29.71
	FOS	FBJ murine osteosarcoma viral oncogene homolog	−29.58
	SNORD91B	Small nucleolar RNA, C/D box 91B	−28
	FOSB	FBJ murine osteosarcoma viral oncogene homolog B	−24.05
	SNORD14D	Small nucleolar RNA, C/D box 14D	−16.61
	TNFSF10	Tumor necrosis factor (ligand) superfamily, member 10	−16.59
	EGR3	Early growth response 3	−16.12
10 most upregulated genes	PHLDA1	Pleckstrin homology-like domain, family A, member 1	11.9
	EDIL3	EGF-like repeats and discoidin I-like domains 3	13.1
	CDH13	Cadherin 13	14.26
	NT5E	5-nucleotidase, ecto (CD73)	14.59
	NRG1	Neuregulin 1	17.49
	FST	Follistatin	23.35
	KIAA1644	KIAA1644	27.67
	FHL1	Four and a half LIM domains 1	28.09
	CCND2	Cyclin D2	28.52
	DUSP4	Dual specificity phosphatase 4	41.23

We used siRNA to investigate whether heregulin upregulation causes aberrant HER3 and Akt phosphorylation. Heregulin-targeting siRNA reduced the level of heregulin mRNA in FaDuCR cells to 10% of that in the siMock control (Fig. 2f). Heregulin knockdown diminished HER3 and Akt phosphorylation in FaDuCR cells to levels lower than those in the control (Fig. 2g). Therefore, heregulin upregulation causes aberrant HER3 and Akt phosphorylation in FaDuCR cells. FaDuCR cells with heregulin knockdown also recovered cetuximab sensitivity. Cetuximab did not decrease the viable counts of FaDuCR cells with siMock, whereas cetuximab significantly decreased the viable counts of FaDuCR cells induced by heregulin-targeting siRNA compared with that of untreated FaDuCR cells (Fig. 2h). We also examined whether exogenous heregulin causes cetuximab resistance in FaDu cells. An in vitro growth inhibition assay demonstrated that cetuximab decreased the viable counts of FaDu cells in a concentration-dependent manner (Fig. 2i). However, exogenous heregulin in the medium induced cetuximab resistance in FaDu cells such that their viable counts were maintained even at 100 μg mL^−1^ cetuximab (Fig. 2i). Furthermore, we examined whether exogenous heregulin can alter the HER3-Akt pathway by immunoblotting. FaDu cells were treated with 10 μg mL^−1^ cetuximab in the presence or absence of 50 ng mL^−1^ heregulin for 1 h, and then harvested for immunoblotting. The addition of heregulin increased HER3 phosphorylation in FaDu cells and maintained Akt phosphorylation in FaDu CR cells treated with cetuximab (Fig. 2j). Interestingly, FaDu cells in the absence of exogenous heregulin decreased HER2 phosphorylation after cetuximab treatment, whereas FaDu cells in the presence of exogenous heregulin maintained HER2 phosphorylation (Fig. 2j). This indicated that HER2 activation depends on EGFR in FaDu cells in the absence of heregulin; in contrast HER2 activation depends on HER3 in FaDu cells in the presence of heregulin. Furthermore, FaDuCR cells were evaluated for sensitivity to HER2 inhibition by the anti-HER2 antibody pertuzumab using the in vitro growth inhibition assay. FaDuCR cells were resistant to either cetuximab or pertuzumab alone, whereas they were sensitive to the combination of both drugs (Fig. 2k). These results indicated that cetuximab inhibits the EGFR-Akt pathway in FaDu cells, whereas heregulin upregulation activates the HER3-Akt pathway responsible for inducing cetuximab resistance. Furthermore, HER3 activation depends on HER2 due to coupling of these receptors in the presence of heregulin.

### Second-generation EGFR-TKI is effective in cetuximab-resistant FaDuCR cells

**Fig. 3:**
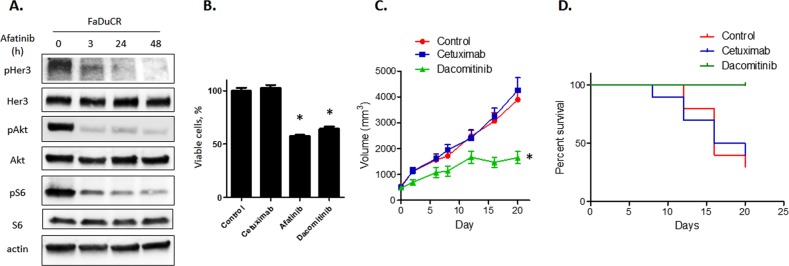
Second-generation EGFR-TKI is effective in cetuximab-resistant FaDuCR cells. **a** FaDuCR cells were treated with 100 nM afatinib for up to 48 h and probed for the indicated proteins. **b** FaDuCR cells were treated with 10 ng mL^−1^ cetuximab, 100 nM afatinib, 100 nM dacomitinib, or vehicle control for 5 d. Cell viability was evaluated using the CellTiter-Glo assay and is shown relative to untreated control cells (mean ± SD of six independent experiments). **c**, **d** In a mouse FaDuCR xenograft model, tumors were treated with cetuximab 40 mg kg^−1^ twice weekly, dacomitinib 15 mg kg^−1^, or vehicle. Each group consisted of 10 mice. Data are mean ± SEM. Tumor growth curves (**c**) and survival curves for tumor-bearing mice in each group (**d**) are shown. *P < 0.05

We explored whether second-generation EGFR-TKI blocked the HER3-Akt signaling pathway in FaDuCR cells. Second-generation EGFR-TKI is a pan-HER family tyrosine kinase inhibitor and may directly or indirectly inhibit HER3 activation^25^. FaDuCR cells were treated with the second-generation EGFR-TKI 100 nM afatinib for up to 48 h and harvested for cell signaling analysis by immunoblotting. Unlike cetuximab (Fig. 2d), afatinib decreased HER3 phosphorylation in FaDuCR cells in a time-dependent manner (Fig. 3a). Afatinib also decreased the phosphorylation of HER3 downstream of Akt and S6 (Fig. 3a). Afatinib blocks the HER3-Akt signaling pathway in FaDuCR cells. Therefore, we assessed whether afatinib and the second-generation EGFR-TKI dacomitinib prevent the proliferation of cetuximab-resistant FaDuCR cells. FaDuCR cells were treated with 100 μg mL^−1^ cetuximab, 10 nM afatinib, or 10 nM dacomitinib for 72 h and their viable counts were determined. In contrast to cetuximab, afatinib and dacomitinib significantly decreased viable cell counts relative to those of the control (Fig. 3b). The efficacy of second-generation EGFR-TKIs was also evaluated in an in vivo mouse xenograft model. FaDuCR-xenografted mice were treated with the vehicle, 40 mg mL^−1^ cetuximab, or 15 mg kg^−1^ d^−1^ dacomitinib. Cetuximab did not prevent tumor growth or prolong survival time (Fig. 3c, d). However, dacomitinib significantly prevented tumor growth and improved survival (Fig. 3c, d). These results suggest that heregulin-mediated, cetuximab-resistant HNSCC may be sensitive to second-generation EGFR-TKIs because these agents block the HER3-Akt signaling pathway.

### HER3 ligand heregulin is aberrantly expressed in HNSCC

**Fig. 4:**
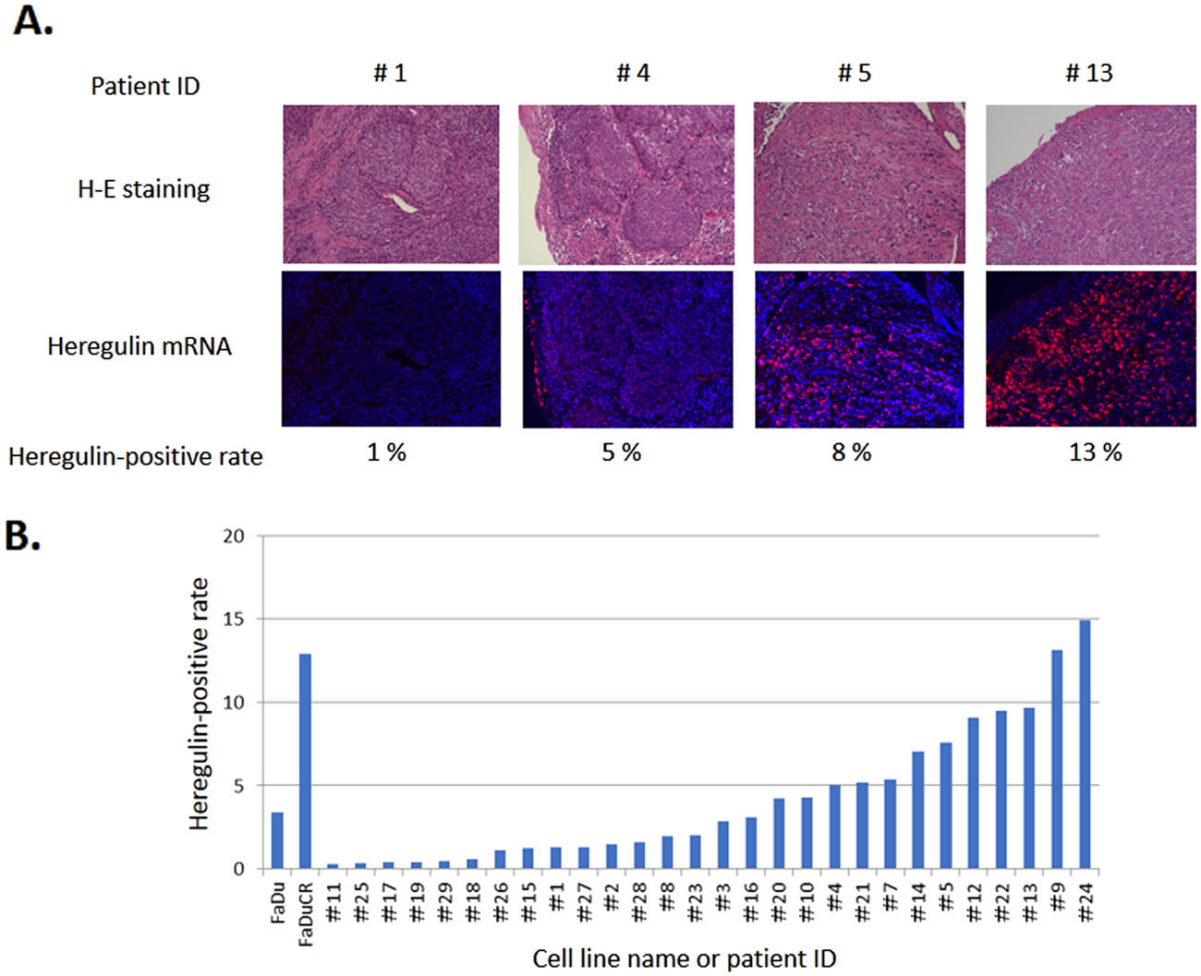
Heregulin mRNA expression in HNSCC tumors. **a** Heregulin mRNA in situ hybridization with H&E staining and heregulin-positive rates. **b** Heregulin-positive rates for FaDu cells, FaDuCR cells, and tumors obtained from patients with HNSCC (*n* = 28)

A cell line-based analysis demonstrated that the HER3 ligand heregulin causes cetuximab resistance in HNSCC. We evaluated heregulin expression in tissue samples from patients with HNSCC who had not received cetuximab-based therapy. Heregulin mRNA expression was detected by ISH, quantitatively evaluated, and found to vary substantially among the 28 tissue samples (range, 0.2–14.9%; median, 2.4%) (Fig. 4a). Tissue samples No. 9 and 24 aberrantly expressed heregulin in a manner equivalent to that observed in cetuximab-resistant FaDuCR cells (Fig. 4b). A patient with No. 9 tumor was diagnosed with recurrent lingual cancer and treated with cetuximab combined with weekly paclitaxel treatments as the second-line chemotherapy. Nevertheless, the tumor was resistant to cetuximab therapy and rapidly progressed within 3 wk. HNSCC with aberrant heregulin expression may be resistant to cetuximab therapy in a manner similar to cetuximab-resistant FaDuCR cells.

## Discussion

The present study results showed that the aberrant expression of the HER3 ligand heregulin induces resistance to the anti-EGFR antibody cetuximab in HNSCC. A subpopulation of tumors obtained from patients with HNSCC aberrantly expressed heregulin in a manner comparable to that of cetuximab-resistant HNSCC FaDuCR cells. Cetuximab-based therapy had limited efficacy in a patient with high heregulin-expressing HNSCC. Therefore, aberrant heregulin expression could induce cetuximab resistance in patients with HNSCC. Poor cetuximab efficacy in patients with HNSCC expressing heregulin at high levels could also be explained by the unfavorable prognostic influence of heregulin. On the contrary, in another cohort consisting of patients with metastatic HNSCC, heregulin mRNA expression was not correlated with survival (Supplementary Fig. 2). Patients with CRC expressing heregulin at high levels were less responsive to cetuximab-based therapy and had shorter progression-free survival (PFS) than those expressing heregulin at low levels^17^. Induction of the heregulin-encoding gene *NRG1* alters CRC and NSCLC cells harboring an EGFR-activating mutation in such a way that they are resistant to EGFR-targeted agents^26,27^. Heregulin might cause resistance to EGFR-targeted therapy in numerous types of cancer. Unlike CRC and NSCLC harboring an EGFR-activating mutation, HNSCC lacks biomarkers for optimizing EGFR-targeted therapy^16^. As high heregulin expression was detected in a subpopulation of HNSCC tumors, the correlation between heregulin expression and the clinical outcome of patients with HNSCC treated with cetuximab merits further investigation.

Despite cetuximab exposure, cetuximab-resistant FaDuCR cells maintained Akt activation mediated by HER3 instead of EGFR. FaDuCR cells were more sensitive than parental FaDu cells to the Akt-specific inhibitor ipatasertib. Mutations in PIK3CA can spontaneously induce the gene and its downstream Akt, causing cetuximab resistance^28^. PIK3CA mutations were detected in patients with CRC resistant to anti-EGFR antibody^29^. These results suggest that the Akt pathway plays a key role in cetuximab resistance. CRC frequently presents with Ras and Raf mutations. Consequently, the Ras/Raf/MAPK pathway contributes to anti-EGFR antibody resistance^16^. However, Ras/Raf mutations are uncommon in HNSCC. Therefore, HNSCC may acquire cetuximab resistance via aberrant Akt mediated by heregulin-dependent HER3 activation. Akt may also be a potential target for cancer therapy (and especially aberrant heregulin expression) in HNSCC.

Unlike the anti-EGFR antibody cetuximab, the pan-HER family tyrosine kinase inhibitors afatinib and dacomitinib may prevent the proliferation of FaDuCR cells. This observation suggests that second-generation EGFR-TKIs could serve as an alternative therapy for patients with cetuximab-resistant and heregulin-expressing HNSCC. A previous study reported that heregulin causes HER3 to couple with HER2 in NSCLC harboring an EGFR-activating mutation. This mechanism induces resistance to first-generation EGFR-TKIs such as erlotinib but not to pan-HER family tyrosine kinase inhibitors such as afatinib^25,26^. In the present study, FaDuCR cells recovered the sensitivity to cetuximab in combination with anti-HER2 antibody pertuzumab. Therefore, the efficacy of pan-HER family tyrosine kinase inhibitor may be due to HER2 kinase inhibition.

Heregulin evaluation by mRNA ISH could detect both cetuximab-resistant and optimal pan-HER family tyrosine kinase inhibitor subpopulations of HNSCC. Afatinib effectively inhibited heregulin expression in the present preclinical model. In contrast, a phase III clinical trial demonstrated significant but minimal improvement in PFS of patients with HNSCC compared to that with methotrexate as a second-line treatment (HR = 0.80; 95% CI = 0.65–0.98)^30^. Subpopulations previously treated with anti-EGFR-targeted antibody were refractory to afatinib (HR = 0.91; 95% CI = 0.70–1.19). In the present study, we did not evaluate heregulin expression after acquired cetuximab resistance. However, 2 of the 28 pre-cetuximab tumors presented aberrant heregulin expression resembling that in FaDuCR cells. The cetuximab resistance mechanism might be heterogeneous in HNSCC. Therefore, afatinib administration would have limited benefit in the treatment of heregulin-mediated, cetuximab-resistant tumors.

The present study is based on a single cell line model and a limited number of clinical samples. To confirm the clinical relevance of the findings of the present study, we must analyze more samples from other HNSCC cohorts known to have acquired resistance to cetuximab. However, HNSCC has limited treatment options compared to that of NSCLC. The molecular mechanisms of HNSCC have not been fully elucidated and their correlations with clinical outcome have not been established. Another report suggested that HNSCC preferentially expresses heregulin compared to other types of cancer^31^. In the present study, we found that heregulin-expressing HNSCC is resistant to cetuximab but not to second-generation EGFR-TKIs. Heregulin may be a promising target of oncotherapy in HNSCC and its clinical relevance requires further research.

## Materials and methods

### Cells and reagents

HNSCC FaDu cell line was obtained from the American Type Culture Collection (ATCC; Manassas, VA, USA). Authenticity of the cells was confirmed by short tandem repeat profile. The cells were maintained in a humidified atmosphere of 5% CO_2_ at 37 °C in RPMI-1640 medium (Sigma-Aldrich Corp., St. Louis, MO, USA) supplemented with 10% fetal bovine serum (FBS) and 1% penicillin–streptomycin. Cetuximab, ipatasertib, afatinib, and dacomitinib were obtained from Selleck Chemicals (Houston, TX, USA).

### Patients

HNSCC tumor specimens were obtained from patients in the Kindai University Faculty of Medicine with the approval of the Institutional Review Board. Written informed consent was procured from each patient.

### In vitro growth inhibition assay

HNSCC cells were plated in 96-well round-bottomed plates at a density of 1 × 10^4^ cells well^−1^. The wells contained RPMI-1640 medium supplemented with 0.5% FBS. After 24 h of incubation, cetuximab, pertuzumab, ipatasertib, erlotinib, and afatinib were added to the wells at various concentrations. After 3 or 5 d of incubation, cell viability was assessed using the CellTiter-Glo luminescence assay (Promega, Madison, WI, USA). Luminescence values are expressed as a percentage of those observed in untreated cells. The 50% inhibitory concentration of each drug was calculated.

### Antibodies and western blotting

HNSCC cells were plated at a density of 1 × 10^6^ on 60-mm Prime Surface plates (Sumitomo Bakelite Co. Ltd., Tokyo, Japan), incubated overnight in medium containing 0.5% FBS, and harvested. Western blotting was performed as previously described^26^. Proteins were probed with antibodies against phospho-AKT, AKT, phospho-HER3, HER3, phospho-HER2, HER2, phospho-S6, and S6 (Cell Signaling Technology, Danvers, MA, USA), phospho-ERK1/2 (Santa Cruz Biotechnology, Dallas, TX, USA), and β-actin (Sigma-Aldrich Corp., St. Louis, MO, USA).

### Comprehensive transcriptome analysis

A comprehensive transcriptome analysis was conducted using the GeneChip Human Transcriptome Array (HTA) v. 2.0 (Affymetrix, Santa Clara, CA, USA). The cRNA was prepared from 100 ng of total RNA and used to generate ssDNA, which was then fragmented and biotinylated. Labeled ssDNA was hybridized for 16–18 h at 45 °C on the HTAs, which were then washed and stained with a streptavidin–phycoerythrin conjugate in Affymetrix Fluidics Station 450 (Affymetrix, Santa Clara, CA, USA). The microarrays were scanned with a GeneChip Scanner (3000 7G; Affymetrix, Santa Clara, CA, USA) according to the manufacturer’s guidelines. The output CEL files were analyzed with Affymetrix Expression Console v. 1.4 (Affymetrix, Santa Clara, CA, USA), which normalizes array signals with a robust multiarray averaging algorithm. The normalized data were analyzed with Transcriptome Analysis Console v. 3.0 (Affymetrix, Santa Clara, CA, USA).

### Reverse transcription and real-time PCR

Total RNA was isolated from HNSCC cells using the RNeasy Mini Kit (Qiagen, Valencia, CA, USA) according to the manufacturer’s instructions. cDNA was synthesized using the high-capacity RNA-to-cDNA kit (Applied Biosystems, Carlsbad, CA, USA) and used in RT-PCR to quantitate heregulin expression. Glyceraldehyde-3-phosphate dehydrogenase (*GAPDH*) was used as the internal control. Expression levels were determined using a standard method with an ABI 7900 HT system and SDS software (Applied Biosystems, Carlsbad, CA, USA).

### siRNA transfection

siRNA against heregulin (siHRG) was derived from ON-TARGET Plus SMARTpool (No. L-004608-01; Dharmacon, Lafayette, CO, USA). It consisted of a mixture of four sets of 21-nucleotide sense and antisense strands. Non-targeting siRNA (siMock) was used as the nonspecific control. HNSCC cells were seeded at 50% confluence in six-well plates, incubated for 24 h in RPMI-1640 medium supplemented with 0.5% FBS, and transfected with siRNAs using Lipofectamine RNAiMax (Invitrogen, Carlsbad, CA, USA). After 72 h, HNSCC cells were harvested for western blotting or in vitro growth inhibition assay.

### In vivo tumor growth inhibition assay

All animal experiments were performed in accordance with the Recommendations for Handling of Laboratory Animals for Biomedical Research in compliance with the Committee on Safety and Ethical Handling Regulations for Laboratory Animal Experiments of Kindai University. The protocol was also reviewed and approved by the Animal Ethics Committee of the Kindai University. FaDu or FaDuCR cells (5 × 10^6^) were subcutaneously injected into the right flank of each female BALB/cAJcl-*nu*/*nu* mouse (CLEA Japan, Tokyo, Japan). When the tumors reached the target volume of 0.2 cm^3^, the mice were randomly assigned to the treatment and control groups. The mice received twice weekly i.p. injections of phosphate-buffered saline (PBS; 100 µL; control), cetuximab (40 mg kg^−1^ body weight in 100 µL PBS), or daily p.o. administration of dacomitinib (15 mg kg^−1^ body weight in 100 µL PBS). Tumor volume and body weight were measured twice weekly without experimenter-blindness. The mice were sacrificed if the tumors became necrotic or grew to a volume of 3.0 cm^3^. Tumor volume was defined as 1/2 × length × width^2^. The T/C ratio was calculated as follows:

1 $$\begin{array}{l}{\mathrm{T:C}} = {\mathrm{100}} \times \left( {{\mathrm{average}}\,{\mathrm{tumor}}\,{\mathrm{volume}}\,{\mathrm{of}}\,{\mathrm{the}}\,{\mathrm{treated}}\,{\mathrm{group}}} \right)\\ {\mathrm{/}}\left( {{\mathrm{average}}\,{\mathrm{tumor}}\,{\mathrm{volume}}\,{\mathrm{of}}\,{\mathrm{the}}\,{\mathrm{control}}\,{\mathrm{group}}} \right)\end{array}$$

### mRNA in situ hybridization

The mRNA expression was evaluated using the QuantiGene ViewRNA ISH cell assay (Affymetrix, Santa Clara, CA, USA) with a human NRG1 probe (NM_001159995; No. VA1-15922). The tumors were fixed in formalin, embedded in paraffin, sliced into 4-µm-thick sections, and stained with a QuantiGene ViewRNA probe set according to the manufacturer’s instructions^32^. The nuclei were counterstained with Hoechst dye. Images were generated using a BZ-X710 microscope (Keyence, Osaka, Japan). Images were acquired and processed in BZ-X Viewer (Keyence) and quantified with ImageJ (National Institutes of Health, Bethesda, MD, USA).

### Statistical analysis

Data were analyzed with SPSS v. 22.0 (IBM Corp., Armonk, NY, USA). For hypothesis testing, an unpaired *t*-test was used for continuous variables and a log-rank test was applied for time-to-event where two-sided *P*-values were obtained. In cases where the outcome variable was time-to-event, Kaplan–Meier curves were constructed. Graphical depictions of the data were generated with GraphPad Prism 5.0 for Windows (GraphPad Software, Inc., La Jolla, CA, USA).

## Supplementary information

Supplemental Figure 1

Supplemental Figure 2
