# Supplementary figures and images for: Aberrant HER3 ligand heregulin-expressing head and neck squamous cell carcinoma is resistant to anti-EGFR antibody cetuximab, but not second-generation EGFR-TKI
Source: Oncogenesis. 2019 Sep 30;8(10):54. doi: 10.1038/s41389-019-0164-9 (PMC6769016; doi:10.1038/s41389-019-0164-9)

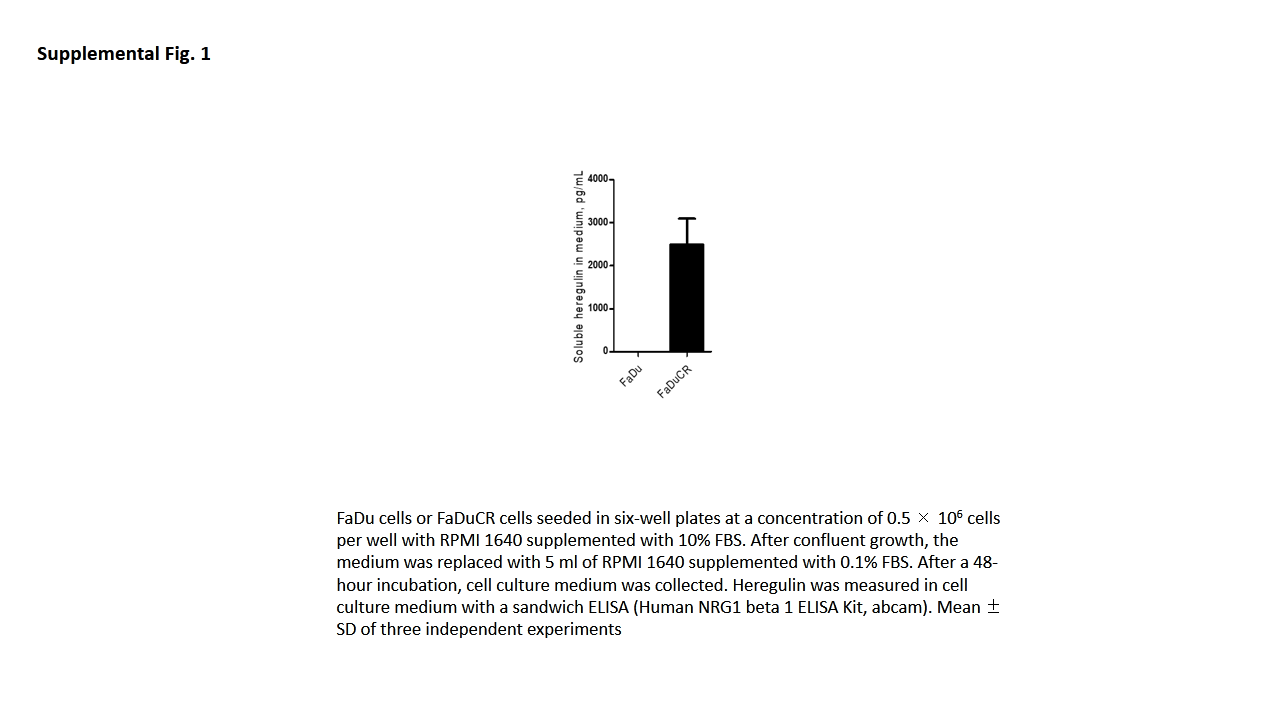

Supplement: Supplementary file 1 — Supplemental Figure 1 [file 41389_2019_164_MOESM1_ESM.tif]

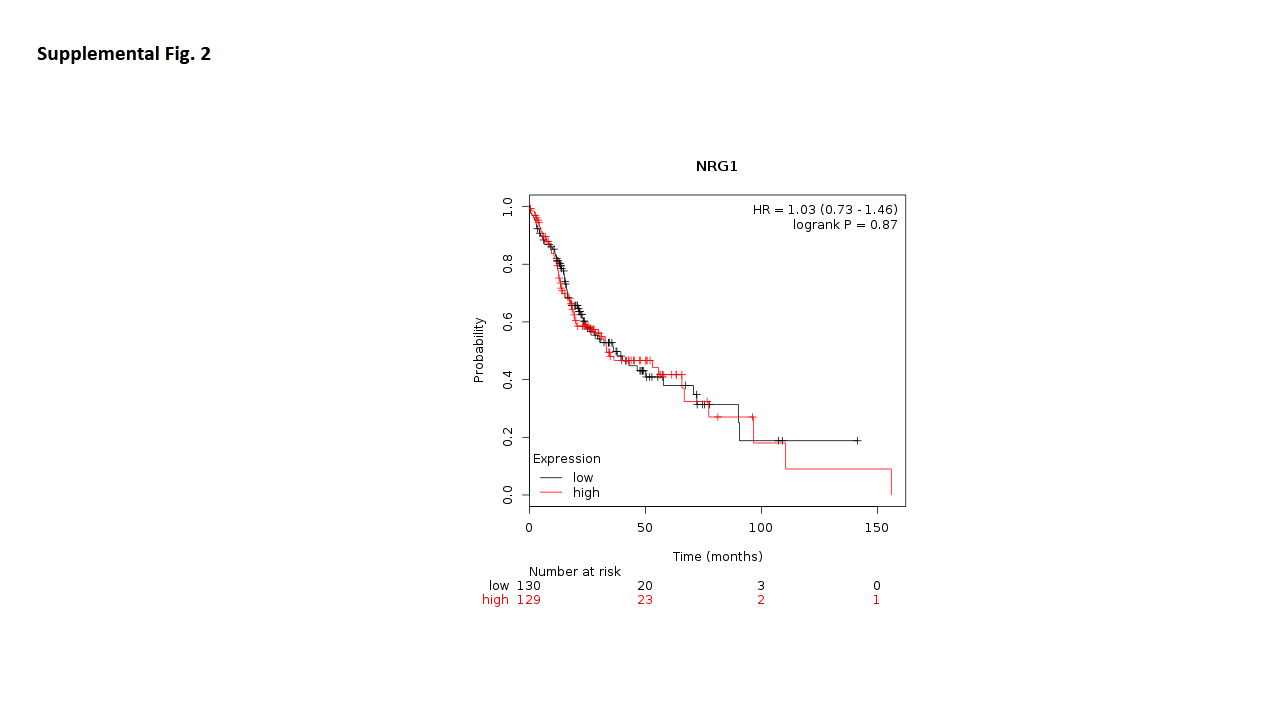

Supplement: Supplementary file 2 — Supplemental Figure 2 [file 41389_2019_164_MOESM2_ESM.tif]
